# Immunomodulatory Effects of Mesenchymal Stem Cells and Mesenchymal Stem Cell-Derived Extracellular Vesicles in Rheumatoid Arthritis

**DOI:** 10.3389/fimmu.2020.01912

**Published:** 2020-08-20

**Authors:** Huan Liu, Ruicen Li, Tao Liu, Leiyi Yang, Geng Yin, Qibing Xie

**Affiliations:** ^1^Department of Rheumatology and Immunology, West China Hospital, Sichuan University, Chengdu, China; ^2^Health Management Center, West China Hospital, Sichuan University, Chengdu, China

**Keywords:** rheumatoid arthritis, mesenchymal stem cells, extracellular vesicles, exosomes, microRNAs

## Abstract

Rheumatoid arthritis (RA) is a chronic autoimmune disease that affects the joints and other organs for which there is currently no effective treatment. Mesenchymal stem cells (MSCs) have therapeutic potential due to their immunomodulatory and differentiation effects. While extensive experimental studies and clinical trials have demonstrated the effects of MSCs in various diseases, MSCs have been found to cause abnormal differentiation and tumor formation. Therefore, extracellular vesicles derived from MSCs (MSC-EVs) are more effective, less toxic, and more stable than the parental cells. MSC-EVs transfer various nucleic acids, proteins, and lipids from parent cells to recipient cells, and thus participate in chronic inflammatory and immune processes. In this review, we summarize the properties and biological functions of MSCs and MSC-EVs in RA. Improvement in our understanding of the mechanisms underlying MSC and MSC-EVs in RA provides an insight into potential biomarkers and therapeutic strategies for RA.

## Introduction

Rheumatoid arthritis (RA) is a prevalent systemic autoimmune disease characterized by progressive joint destruction, and 50% of RA patients also have extra-articular involvement, including the heart, lungs, eyes, and blood (1, 2). Globally, the overall incidence of RA is 40/100,000 people per year, with a prevalence of ~0.24% (3, 4), which is significantly higher in women (5). The etiologies and pathogenesis of RA have been extensively studied, wherein genetic susceptibility (i.e., *HLA DR1, TRAF1*, and *STAT4*), epigenetic modification (i.e., DNA methylation, miR146a, and miR155), and environmental factors (i.e., smoking, obesity, periodontitis, and vitamin D deficiency) have been found to promote the loss of immune tolerance, resulting in this disorder (1, 6–8). However, the precise mechanism underlying RA is complex and has not yet been elucidated. Currently, RA treatments, including glucocorticoid, immunosuppressants, and biological agents, are non-specific with an inadequate efficacy, severe adverse reactions, and even life-threatening toxic effects (7, 9).

Mesenchymal stem/stromal cells (MSCs) are a class of stem cells with self-renewal and multipotent properties that are widely available. As such, extensive clinical research has focused on the effects of MSCs in tissue regeneration and protection against injury via the replacement of damaged cells (10, 11). Subsequently, the evidence is increasingly indicating that MSCs play an immunomodulatory role primarily through the release of extracellular vesicles (EVs) and paracrine factors (e.g., growth factors, hormones, and cytokines) (11, 12). MSCs originate from many types of tissues, including bone marrow (BM), adipose tissue (AT), umbilical cord (UC), cord blood (CB), peripheral blood, dental pulp, liver, and the synovial membrane (12, 13). Generally, MSCs mostly express CD73, CD90, and CD105; however, these surface markers cannot be used to discriminate the source of MSCs. In contrast, MSCs negatively express CD14, CD34, CD45, and HLA-DR. MSCs can escape T cell recognition and exhibit low immunogenicity (14–17).

EVs are a group of lipid-bound vesicles that are released by various cells and play an essential role in the transfer of information between adjacent or distant cells. According to their origin, secretion mechanisms, and properties, EVs are divided into apoptotic bodies, microvesicles (MVs), and exosomes. Apoptotic bodies (50–5,000 nm) are released by dying cells into the extracellular space, and contain intact organelles, chromatin, and small amounts of glycosylated proteins. MVs (100–1,000 nm) originate from plasma membranes. Exosomes (30–150 nm) are formed by the intraluminal buds of multivesicular endosomes (MVEs) (18, 19). Due to the limitations of separation technologies, small EVs (sEVs) (50–200 nm) are commonly used in experimental studies (20). Among the different cells known to produce EVs, MSCs are one of the most prolific cells (21). Phenotypically, MSC-derived sEVs also express the MSC markers CD73, CD90, and CD105, but not CD14, CD34, or CD11b (17). The functions of MSC-EVs are similar to those of MSCs, although the latter are more stable, safe, less toxic, and are able to pass the blood-brain barrier, thus reducing their propensity to trigger immune responses (22–24). MSC-EVs transfer nucleic acids, including DNA, mRNA, and microRNA (miRNA); lipids; proteins; and surface receptors from donor cells to specific recipient cells, thereby protecting signaling molecules from enzymatic degradation during transport. MSC-EVs fuse with the recipient cell membrane either by directly fusing with the plasma membrane, fusing with the endosomal membrane after endocytosis, or by directly binding to the receptor of recipient cells, and then participate in physiological and pathological processes (25–27).

In recent years, studies have shown that MSCs and MSC-EVs may be effective in RA, highlighting their potential immunomodulatory effects. In this review, we aim to discuss recent advances in the use of MSCs and MSC-EVs for the treatment of RA.

## Immunomodulatory Effect of MSCs in RA

In the past decade, MSC transplantation (MSCT) has been found to be effective in the treatment of RA by reducing joint inflammation, bone erosion, and destruction and alleviating the formation of pannus via immune regulation, anti-inflammation, and differentiation (28, 29). MSCs mainly interact with both innate and adaptive immune cells to modulate immune responses in RA.

MSCs may regulate the proliferation, differentiation, and function of T cells and reduce the production of pro-inflammatory factors. In mouse models with collagen-induced arthritis (CIA), the administration of human AT-derived MSCs (AT-MSCs) inhibited the differentiation of activated CD4+ T cells into T helper (Th) 17 effector cells producing interleukin (IL)-17, but induced the generation of T regulatory cells (Tregs) that secrete IL-10 and negatively regulate the immune response (30). Similar beneficial effects have been reported in RA animal models using various MSC treatments (28, 31, 32). The effects of MSCs on Th17/Treg cell balance have been attributed to various soluble molecules, including indoleamine 2,3-dioxygenase (IDO), IL-10, prostaglandin E2 (PGE2), and nitric oxide (NO), and to the transfer of organelles (32, 33). For example, after co-culturing healthy mice bone marrow-derived MSCs (BM-MSCs) and Th17 from peripheral blood mononuclear cells (PBMCs) of RA patients, the proliferation of Th17 cells and production of IL-17 was inhibited by transferring mitochondria from BM-MSCs to Th17 cells. Simultaneously, mitochondrial transfer from the BM-MSCs of healthy donors was higher than that from the synovium-derived MSCs of RA patients (32). T follicular helper (Tfh) cells, a subset of CD4+ T cells, may help in immunoglobulin affinity maturation and generate live plasma cells and memory B cells (34, 35). Liu et al. found that the number of circulating Tfh cells increased, and was positively correlated with the disease and anti-cyclic citrullinated peptide antibody levels in RA patients (36). Subsequently, they further demonstrated that allogeneic UC-derived MSCs (UC-MSCs) suppressed the proliferation and function of Tfh cells via IDO production, which may be induced by interferon (IFN)-γ *in vivo* and *in vitro*, thereby ameliorating the progression of CIA (37). Endoplasmic reticulum (ER)-stressed MSCs could reduce the number of circulating Tfh cells via higher PGE2 binding with EP2/EP4 and increased IL-6 levels (38).

B cells mainly produce autoantibodies, including rheumatoid factor (RF) and anti-citrullinated protein antibodies (ACPAs), but also secret cytokines and act as antigen-presenting cells to promote T cell activation in RA (39). MSCs from healthy donors have been found to suppress B cell proliferation and anti-ACPA and RF production (29, 40). However, the mechanism underlying B cell regulation by MSCs in RA remains unclear. Currently, autologous MSCs injection has been considered to decrease B cell responses by reducing the levels of the B-cell activation factor (BAFF), a proliferation-inducing ligand (APRIL), and BAFF receptors (29). In comparison, in an *in vitro* experiment, BM-MSCs from RA patients co-cultured with B cells from PBMC of healthy donors supported B cell survival, by a mechanism that may not be correlated with BAFF (41). This may be because of the conditional complexity of *in vitro* and *in vivo* experiments. In addition, the inhibition of MSCs on Tfh cells also indirectly affected the proliferation and differentiation of B cells (37).

Dendritic cells (DCs), macrophages, and natural killer (NK) cells are important members of the innate immune response and are regulated by MSCs in various diseases (42–44). However, their interaction with MSCs is scarcely studied in RA. Shin et al. demonstrated that MSCs inhibited the activation of M1-type macrophages and induced the generation of M2-type macrophages via the tumor necrosis factor (TNF)-α-mediated activation of cyclooxygenase-2 (COX-2) and TNF-stimulated gene-6. This was accompanied by the negative regulation of the nucleotide-binding domain, leucine-rich repeat pyrin 3 (NLRP3) inflammasome-mediated IL-1β secretion, and caspase-1 production in macrophages through an IL-1β feedback loop (45). In addition, MSCs from systemic juvenile idiopathic arthritis patients were found to inhibit the differentiation of monocytes to DCs and suppress NK cell activation (46). Li et al. found that the combination of tolerogenic DCs and MSCs had a synergistic immunosuppressive effect on CIA mice by polarizing Th cells and inhibiting pro-inflammatory cytokines (47).

## Clinical MSC Trials in RA

In recent years, clinical research on the use of MSC therapy for the treatment of RA has increased. The first randomized clinical trial (RCT) using allogeneic expanded AT-MSCs (Cx611) for RA treatment was conducted in 2011 as a multicenter, single blind, and placebo-controlled phase Ib/IIa clinical trial. A total of 53 refractory RA patients were enrolled and assigned to three cohorts with different doses (1, 2, or 4 million cells/kg) and a placebo cohort, to evaluate the safety and tolerability of Cx611. The results indicated that the infusion of Cx611 was generally well-tolerated. One patient with dose-limiting toxicity (DLT) presented lacunar infarction. Most adverse events (AE) were mild or of moderate intensity. Although the most common symptoms were fever and infection, it was difficult to discern whether these were symptoms or simply side effects of Cx611 (48). In a phase Ia RCT investigating the efficacy and safety of the intravenous infusion of human CB-derived MSCs (hCB-MSCs), 9 RA patients were divided equally among three groups, each receiving a single intravenous infusion of hCB-MSCs at different dosages. No short-term AE or DLT were reported 4 weeks after infusion. Moreover, the DAS 28 (28-joint disease activity score) was significantly decreased, pro-inflammatory cytokines were reduced, and IL-10 levels were increased 24 h after infusion (49). Similarly, a single-center RCT selected 30 RA patients with knee involvement to receive either intra-articular knee autologous BM-MSCT (*n* = 15) or normal saline (*n* = 15). Following the transplantation of 40 million autologous BM-MSCs, although no statistically significant results between the two groups were noted in the majority of the outcome measures, favorable effects on joint inflammation symptoms were observed, with an improved standing time in the MSCT group (*p* = 0.02). Moreover, MSCT treatment helped to reduce the dosage of MTX and prednisolone during the initial 6 months of follow-up, although not after 1 year. Importantly, no AEs were observed after MSC administration or during follow-up (50). Similarly, a clinical trial in Iran investigated whether injections of autologous BM-MSCs relieved the symptoms of refractory RA patients (51). These clinical trials found that MSC therapy for RA, especially refractory RA, is safe, well tolerable, and effective.

## Immunomodulatory Effects of MSC-EVs in RA

**Figure 1:**
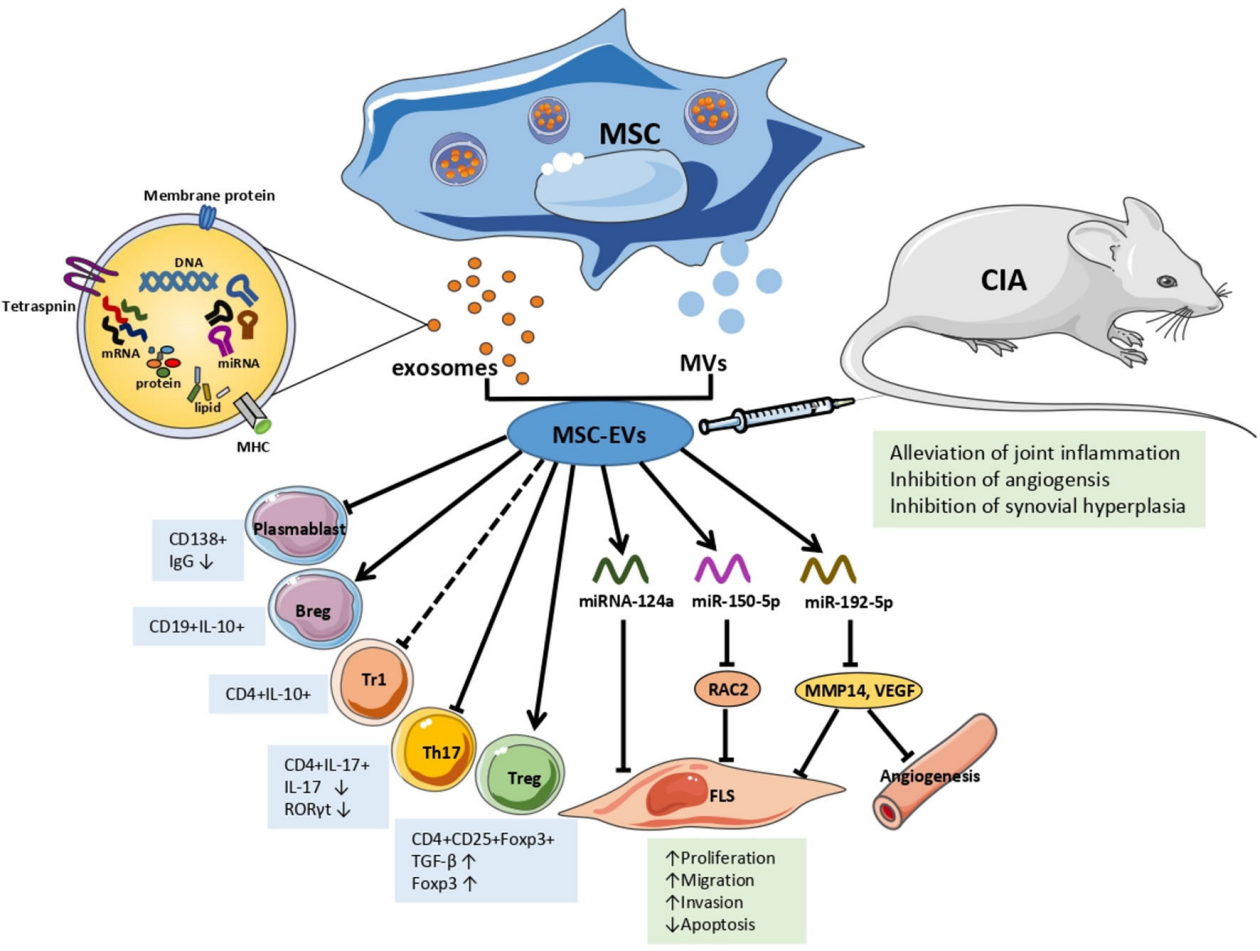
The effects and mechanisms of MSC-EVs in RA. Arrows indicate activation or induction, T-bars indicate inhibition, dotted T-bars indicate inconsistent result, with Tr1 cells increasing *in vitro* but decreasing *in vivo*. MSC, mesenchymal Stem Cell; MVs, microvesicles; EVs, extracellular vesicles; Tr1, T regulatory type 1 cells; Th 17, T helper 17 effector cells; Treg, T regulatory cells; FLS, fibroblast-like synoviocytes; IgG, immunoglobulin G; TGF-β, transforming growth factor β; IL, interleukin; miRNA/miR, microRNA; RAC2, ras-related C3 botulinum toxin substrate 2; MMP14, matrix metalloproteinase 14; VEGF, vascular endothelial growth factor; CIA, collagen-induced arthritis mice model.

Recent studies have indicated that the mechanisms underlying the interaction between MSC-EVs and recipient cells are not unique in terms of their physiological or pathological processes in RA (Figure 1). Ma et al. found that both human UC-MSCs and the EVs secreted by them inhibited the proliferation of T cells, promoted T cell apoptosis, decreased RORγ levels, increased Foxp3 levels, and regulated the balance of Treg/Th17 cells in *in vitro* and *in vivo* experiments, resulting in delayed radiological progression and synovial hyperplasia inhibition (52). Notably, the partial effect of MSC-EVs was different from that of parental MSCs in RA. MSC-exosomes increased the number of Treg, whereas MSCs did not. And MSCs were more capable of reducing the number of CD4+IFN-γ+ T lymphocytes. Compared with parental BM-MSCs and MVs, the exosomes increased the number of Treg cells. In addition, this study showed that MSC-exosomes inhibited plasmablasts but generated Breg cells (53).

Based on the fact that EVs are able to transfer information to recipient cells, subsequent studies characterized the mechanisms by which EVs, particularly through miRNAs, are involved in RA. Chen et al. were the first to report that BM-MSC-EVs transferred miR-150-5p to the joint cavity. Compared with osteoarthritis patients, the expression levels of miR-150-5p in the serum, synovial tissues, and fibroblast-like synoviocytes (FLS) of RA patients were significantly decreased, whereas the expression levels of matrix metalloproteinase (MMP) 14 and vascular endothelial growth factor (VEGF) were increased. MiR-150-5p was effectively transfected into BM-MSCs *in vitro* and transferred by exosomes to RA-FLS. MSC-exosomal miR-150-5p suppressed the expression of the target genes MMP14 and VEGF by directly binding to their 3'-UTRs, thereby reversing the migration and invasion of RA-FLS and HUVEC tube formation induced by pro-inflammatory factors, including IL-1β, transforming growth factor β (TGF-β), and TNF-α. *In vivo*, the effect of MSC-exosome-miR-150-5p injection was consistent with those mentioned above, wherein MSC-exosome-miR-150-5p inhibited angiogenesis and alleviated joint inflammation (54). Recently, miR-192-5p expression was found to be decreased in human RA-FLS, wherein a dual luciferase reporter gene assay showed that miR-192-5p directly targeted and negatively regulated ras-related C3 botulinum toxin substrate 2 (RAC2). In a CIA rat model, MSC-exosomal miR192-5p was transferred to the synovial tissue via the blood circulation after injection, and significantly reduced the levels of RAC2, decreased the clinical score, and suppressed synovial hyperplasia and joint destruction compared with rats injected with BM-MSCs-exosome-NC. Additionally, MSC-exosome-miR-192-5p inhibited the levels of pro-inflammatory cytokines, including PGE2, IL-1β, and TNF-α, in synovial tissues and serum, and reduced the release of NO and inducible NO synthase (iNOS) in the sera of CIA rats (55). Another *in vitro* experiment found that the exosome number and miRNA-124a levels increased in MH7A cells (RA-FLS cell lines) after co-culturing MH7A with human MSC-EV. With miRNA-124a (hMSC-124a-EV) overexpression, the proliferation of MH7A was inhibited by hMSC-124a-EV and hMSC-EV compared with that in the control group. However, the cells were blocked in the G0/G1 and S phases, respectively. The invasion and migration of MH7A were also suppressed, while apoptosis was promoted. Moreover, the effect of hMSC-124a-EV treatment was more marked than that of hMSC-EV (56).

## Prospects and Challenges in the Clinical Application of Mscs and MSC-EVs in RA

With an increasing number of studies, MSCs have been found to play an immunomodulatory role in numerous autoimmune diseases through the production of soluble factors, and the transfer of EVs containing messaging molecules (11, 57–59). In addition to immune regulation, MSCs can induce osteogenic and chondrogenic differentiation, and regulate inflammatory factors, highlighting it as a promising therapy for RA. Currently, most clinical trials of MSCT therapy for RA have focused on refractory RA patients who have not responded to traditional disease modifying antirheumatic drug (DMARDs) therapy, without any serious AEs associated with MSCT treatment. However, the use of MSCs in therapeutic treatments still faces many challenges. Several studies have found that MSCs are associated with carcinogenic risk when injected in animal models (60–63). Allogeneic MSCs have an immunosuppressive effect on tumor cells, allowing them to evade detection and destruction by the adaptive immune regulatory system via the action of CD8+ T cells, leading to the growth of allogeneic tumor cells (61). MSCs could also secrete VEGF to induce angiogenesis (62), contributing to tumor stroma formation, and favor tumor cell proliferation, invasion, and migration (60). The immunosuppressive effects of MSCs in CIA are also debatable. While MSCs can inhibit anti-CD3-induced T-cell proliferation *in vitro*, they do not affect T cell proliferation nor the development of CIA (64). Factors including the type of MSCs, culture conditions, treatment time, number of injected cells, injection route, and treatment regimen can lead to different results. A recent study compared the effects of three different types of MSCs infused into CIA mice and found that the most effective treatment was UC-MSCs, followed by BM-MSCs (65). The efficacy of allogeneic and autologous MSCs remains debatable, Rozier suggests that autologous MSCs may be involved in the physiopathology of systemic sclerosis (66). Therefore, RCT are necessary to compare the efficacy and safety of autologous and allogeneic MSC therapy in RA. BM-MSCs from RA patients were also found to promote Th17 cell activation and expansion via caspase 1 activation (67). In addition, different conditions also influence the effect of MSCs. For example, epigenetically-modified MSCs (combination of hypomethylating agents and histone deacetylase inhibitors) have a high immunoregulatory effect in RA (68). Consequently, determining how long the immunomodulatory effects of MSCs last will need to be solved in clinical practice, the results of which could provide a theoretical basis and support for their use in the treatment of RA.

Compared with MSC treatment, which may cause abnormal differentiation and tumor formation, MSC-EVs are more effective, stable, and safer in alleviating inflammation of CIA, with broader prospects. EVs carry numerous DNAs, RNAs, proteins, and lipids from MSCs and transfer them to the recipient cells. Due to several advantages, including the ability to pass the blood-brain barrier and their low immunogenicity, EVs are natural carriers for drugs and exogenous nucleic acids, which can be loaded in donor cells before being released into the extracellular environment (69, 70). More importantly, using EVs to transfer miRNAs can prevent these from being degraded, allowing miRNAs to negatively regulate target protein expression at the post-transcriptional level. In addition to their treatment potential, several studies have reported that miRNAs secreted by MSC-EVs (MSC-EV-miRNAs) regulate diverse signaling pathways by targeting specific proteins, thereby influencing the development of RA. Therefore, MSC-EV-miRNAs are potential biomarkers for use in novel cell-free therapeutic strategies for RA.

Although MSC-EVs have been used in preclinical RA studies, several issues still remain unsolved. Firstly, when MSCs from different tissues are in distinct differentiation states, the content and types of molecules assembled by EVs may be different, thereby affecting their function at recipient cells and causing changes to physiological processes. MSC-EV miRNAs do not randomly enter EVs, however, the sorting mechanism by which cells are adjusted and selected from maternal cells is unknown. Generally, hundreds of differentially expressed miRNAs could be found in MSC-EVs by sequencing or microarray assays, however, no studies have performed miRNA expression profiling on MSC-EVs between RA and healthy individuals. Currently, dozens of miRNAs have been reported to affect the proliferation and function of FLS, previous studies also focused on the effect of MSC-EV-miRNAs on FLS, further studies on other cells are also needed. Additionally, whether the complex regulatory network of miRNAs and their target genes may trigger other diseases remains unclear and needs further study. Secondly, MSC-EVs secrete many other signaling molecules. Eirin et al. (71) integrated transcriptomic and proteomic analyses and found that the proteins, transcription factors, and translational regulators derived from MSC-EVs are involved in the mechanism of tissue repair in the recipient cell. Further studies on the interactions of the molecules that affect RA are necessary. Thirdly, EVs are separated in different ways, without standards, and in a time-consuming manner. Although commercial exosome extractants are currently being used, they contain non-exosome contaminants, such as lipoproteins, which need to be purified. Lastly, the findings presented here will need to be replicated on a large scale in clinical trials to assess the safety, effectiveness, and persistence of MSC-EVs in RA patients.

## Author Contributions

HL, RL, TL, and LY wrote sections of the manuscript. QX and GY critically revised the manuscript. QX reviewed and approved the version to be published. All authors contributed to manuscript revision, read, and approved the submitted version.

## Conflict of Interest

The authors declare that the research was conducted in the absence of any commercial or financial relationships that could be construed as a potential conflict of interest.
